# Self-Printing on Graphitic Nanosheets with Metal Borohydride Nanodots for Hydrogen Storage

**DOI:** 10.1038/srep31144

**Published:** 2016-08-03

**Authors:** Yongtao Li, Xiaoli Ding, Qingan Zhang

**Affiliations:** 1 School of Materials Science and Engineering, Anhui University of Technology, Maanshan 243002, China

## Abstract

Although the synthesis of borohydride nanostructures is sufficiently established for advancement of hydrogen storage, obtaining ultrasmall (sub-10 nm) metal borohydride nanocrystals with excellent dispersibility is extremely challenging because of their high surface energy, exceedingly strong reducibility/hydrophilicity and complicated composition. Here, we demonstrate a mechanical-force-driven self-printing process that enables monodispersed (~6 nm) NaBH_4_ nanodots to uniformly anchor onto freshly-exfoliated graphitic nanosheets (GNs). Both mechanical-forces and borohydride interaction with GNs stimulate NaBH_4_ clusters intercalation/absorption into the graphite interlayers acting as a ‘pen’ for writing, which is accomplished by exfoliating GNs with the ‘printed’ borohydrides. These nano-NaBH_4_@GNs exhibit favorable thermodynamics (decrease in *∆H* of ~45%), rapid kinetics (a greater than six-fold increase) and stable de-/re-hydrogenation that retains a high capacity (up to ~5 wt% for NaBH_4_) compared with those of micro-NaBH_4_. Our results are helpful in the scalable fabrication of zero-dimensional complex hydrides on two-dimensional supports with enhanced hydrogen storage for potential applications.

A challenge for the widespread use of hydrogen in transportation and in backup and stationary power generation is the compact and safe storage of hydrogen in a solid-state medium^1^,^2^,^3^,^4^,^5^. Many materials have been extensively investigated, including metal hydrides, complex hydrides and high surface area adsorbents such as carbon nanomaterials, zeolites and metal–organic frameworks (MOFs)^6^,^7^,^8^,^9^,^10^,^11^,^12^,^13^; unfortunately, none of the materials has yet met the technical requirements for practical applications^14^. Sodium borohydride, NaBH_4_, with its high hydrogen-containing capacity (up to 10.8 wt% in gravimetric density), has been considered one of the most promising candidates for hydrogen storage in the solid-state^14^,^15^; however, the large standard enthalpies for decomposition and the high kinetic barrier for de-/re-hydrogenation are the two main challenges in this field. The solution to the first challenge is to decrease the thermodynamic stability of NaBH_4_; the solution to the second challenge is to improve the de-/re-hydrogenation kinetics and reversibility^16^.

Much attention has been focused on developing routes for obtaining borohydride nanostructures because their high surface area, abundant grain boundaries/defects and short diffusion distances would facilitate hydrogen desorption and absorption at a faster rate and at a lower temperature than those of the bulk structures^17^,^18^,^19^,^20^,^21^,^22^. Two strategies for reducing the particle size to the nanoscale have been proven effective. The first such approach is mechanical milling (top-down). However, this approach suffers from a wide size distribution of particles (ranging from 2 nm to 10 μm) and from the inevitable particle agglomeration driven by the high surface energy, both of which make it difficult to produce dramatic size effects^23^,^24^.

The second approach is a bottom-up solvothermal route that can solve the issues associated with the first approach to some extent^25^,^26^,^27^,^28^,^29^. For example, Ngene *et al*.^26^ impregnated NaBH_4_ into nanoporous carbon to decrease the nanoparticles aggregation for obtaining much faster desorption kinetics; however, the unacceptable dead mass contributed by the nanosupports reduced the storage efficiency. To address this issue, Meganne *et al*.^27^ adopted a core-shell strategy for restricting the NaBH_4_ particles to a size between 10 and 200 nm using a nanothick Ni-shell coating. Later, Li *et al*.^28^ proposed a reorganized route to obtain ultrathin polymer-capped NaBH_4_ nanoparticles with a size greater than 50 nm. An average diameter of ~45 nm has been recently achieved for NaBH_4_ nanoparticles wrapped by graphene via a wet chemistry method plus de-/re-hydrogenation treatments^29^. Unfortunately, these dissipative nanostructures from multiple-step methods are often randomly reorganized, which provides uncontrolled regularity and location (i.e., the hydrides usually exist simultaneously on both the internal and external surfaces of the scaffolds) and a broad size distribution. In particular, achieving sub-10 nm borohydrides with a homogeneous size distribution and acceptable “dead mass” contributed by the nanosupports is impossible through the existing methods. Indeed, to the best of our knowledge, such borohydrides have not actually been realized in previous studies because of their exceedingly strong reducibility/hygroscopicity and complicated elemental composition. Recently, it was shown that the congeneric LiBH_4_ nanostructures can be uniformly reduced to the size of 10–40 nm by physical vapour deposition (PVD)^22^. But this method is conditioned by adopting the one-dimensional organic coordination polymers containing borohydride groups as precursors, which makes it difficult to be extended to other borohydrides synthesis. Hence, developing a novel method to synthesize ultrasmall NaBH_4_ nanoparticle with excellent dispersibility to establish the essential nanoscale effects is imperative but challenging. Achievement of this goal may also open a door to the nanostructured material designs required to transform the performance of hydrogen storage materials to levels at which they are practical for the thermodynamics and kinetics events.

Here, we present a simple and facile solvent-free, mechanical force-induced self-printing (MFSP) method for the synthesis of ultrasmall (~6 nm) NaBH_4_ nanodots with excellent dispersibility that can self-assemble on multi-layered graphitic nanosheets (nano-NaBH_4_@GNs). We discover this technique serendipitously while examining the positive effects of graphite on the metathesis of LiBH_4_ with NaCl for preparing NaBH_4_ nanocrystals. The ‘printing’ process was induced by the intercalation of NaBH_4_ nanocrystals newly formed via the metathesis reaction of LiBH_4_ and NaCl precursors into the graphite interlayers and by the interaction between the GNs and borohydride. As a consequence of this process, the bottom-up MFSP of NaBH_4_ nanocrystals with well-controlled distribution and size as a ‘dye’ wrote onto the *in situ* exfoliated GNs as a ‘paper board’ was achieved through a one-pot mechanical milling process for the first time. The obtained GNs-supported monodisperse NaBH_4_ nanodots exhibit exceptionally favorable thermodynamics, rapid kinetics and stable nanostructures with better reversibility than their microscale counterparts.

## Results

### Synthesis of nano-NaBH_4_@GNs by MFSP

**Figure 1:**
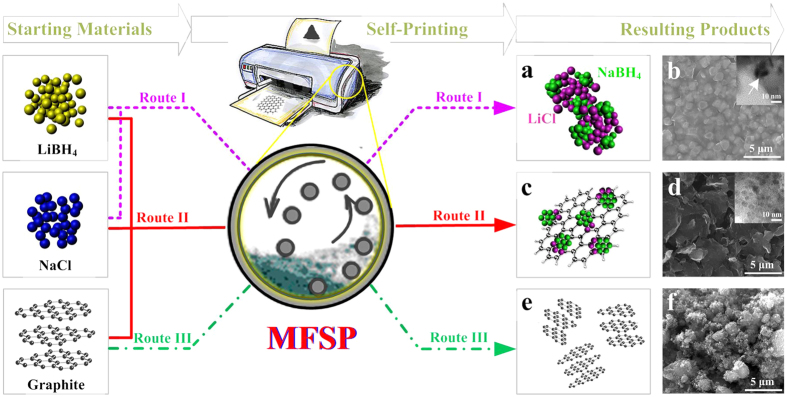
Mechanical‒force‒driven self-printing (MFSP) of the NaBH_4_ nanodots self-assembled on *in situ*‒prepared GNs. Route I: LiBH_4_ and NaCl as starting materials; Route II: LiBH_4_, NaCl and graphite as starting materials; Route III: only graphite as a starting material. (**a,c,e**) Schematics and (**b,d,f**) SEM images of the products obtained via Routes I, II and III after ball milling, respectively. The insets in (**b,d**) are the HRTEM images of the resulting products of Routes I and II after ball milling, respectively.

A typical MFSP preparation is illustrated in Fig. 1. Similar to 3D printing, the raw materials were introduced directly and composites with the desired and reproducible nanostructures were subsequently produced. As an example, commercial LiBH_4_, NaCl and graphite were used as starting materials without purification. Mechanically milling the LiBH_4_ and NaCl mixture in a 1:1 molar ratio (Route I) resulted in the formation of NaBH_4_ nanocrystals (NCs) via a metathesis reaction according to Eq. (1) (Fig. 1a).


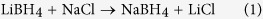


This reaction was demonstrated by the micrographic changes between Fig. 1b and Supplementary Fig. S1, whereby the milled sample exhibits smooth and connective features that differ substantially from the initial cubic and discrete shape before ball milling. The high-resolution transmission electron microscopy (HRTEM) images in the inset of Fig. 1b and Supplementary Fig. S2 further show the milled products for Route I, demonstrating that NaBH_4_ NCs larger than 10 nm are embedded in the LiCl by-product matrix. Interestingly, when the ternary mixture of LiBH_4_, NaCl and graphite was ball-milled using the same procedure (Route II), an encouraging morphology was obtained whereby the resulting products of NaBH_4_ NCs and LiCl were uniformly loaded onto the surfaces of the layered graphite (Fig. 1c). Figure 1d and Supplementary Fig. S3 clearly exhibit an edge-corrugated layered structure for the milled products of Route II, and its HRTEM image in the inset of Fig. 1d further shows nearly monodisperse nanodots smaller than 10 nm uniformly distributed on the exfoliated graphite. These results strongly indicate that when driven by mechanical forces, the graphite tends to exfoliate into a few-layered structure for borohydride anchoring. Note that, this process involved in Route II mimics that used in a ‘dye inject printer’, providing the proof-of-concept for MFSP whereby the borohydride nanocrystals acting as a ‘dye’ is uniformly printed on the exfoliated few-layered graphitic ‘paper board’.

In marked contrast, in the absence of LiBH_4_ and NaCl, the exact same method was selected for ball milling graphite (i.e., Route III), which simply showed an irregular, coarsened morphology without the layered structure obtained from Route II (Fig. 1e,f). This drastic difference highlights the role of LiBH_4_ and NaCl or their resulting products in the exfoliation of graphite except for the mechanical-force-driven effect. To further clarify this effect, the sole LiBH_4_, NaCl, LiCl or NaBH_4_ (micrometer-grade) in the same ratio were individually added into the graphite for ball milling, but none of them exhibited the favorable layered structures, even though some slightly platelet-like structures were obtained for the milled graphite with borohydrides (Supplementary Fig. S4). These results clearly indicate that the metathesis reaction of LiBH_4_ with NaCl, i.e., the *in situ* formation of fine NaBH_4_ NCs, is also a key factor for the exfoliation of graphite, the reason for which will be discussed later.

### Structural and morphological features of nano-NaBH_4_@GNs composites

**Figure 2:**
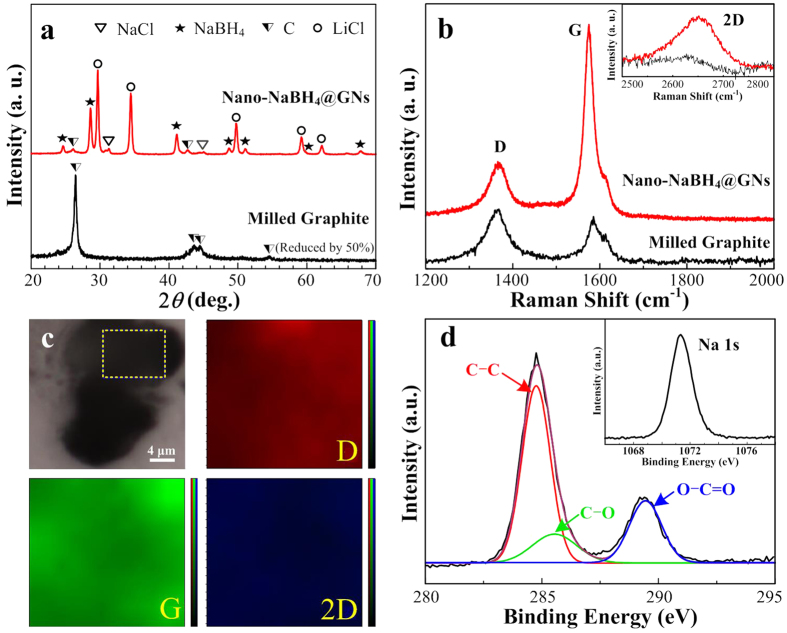
Structural characterization of the nano-NaBH_4_@GNs and milled graphite. (**a**) XRD patterns for the nano-NaBH_4_@GNs and milled graphite; (**b**) Raman spectra showing the *D* and *G* peaks of the nano-NaBH_4_@GNs and milled graphite; their 2*D* peaks are also shown; (**c**) Optical image and corresponding Raman peak intensity mappings of the *D* band, *G* band and 2*D* band for the marked square area of the nano-NaBH_4_@GNs; and (**d**) high-resolution scan of C1s XPS spectrum for the nano-NaBH_4_@GNs; and the corresponding inset shows its Na1s XPS spectrum.

Figure 2 shows the structural and morphological features of nano-NaBH_4_@GNs obtained via Route II, i.e., monodispersed nanodots containing NaBH_4_ and LiCl on the exfoliated graphitic nanolayers. The X-ray powder diffraction (XRD) and Raman patterns for the milled graphite obtained via Route III are also presented for reference. In the case of the milled graphite, typical diffraction peaks were observed at 26.4°, 43.6°, 44.2° and 54.6° in Fig. 2a, again reflecting that milling alone does not change the graphite structure. Interestingly, for nano-NaBH_4_@GNs, only two broader and weaker Bragg peaks originating from the exfoliated graphite were observed at 25.6° and 42.3°, in good agreement with previously reported results for reduced graphene oxide (rGO)^30^,^31^,^32^,^33^. To some extent, this obvious decrease in the 2*θ* value suggests that the solid-state exfoliation of milled graphite occurred under the improved effect of the *in situ* formed NaBH_4_ and LiCl.

We subsequently used Raman spectroscopy to further examine the difference between the carbon structures. The Raman spectra show characteristic peaks of the *D* (at ~1330 cm^−1^), *G* (at ~1595 cm^−1^) and 2*D* (at ~2652 cm^−1^) bands (Fig. 2b), which also agrees well with the reported results for rGO^31^. The *D* peak, arising from the breathing mode of K-point phonons of A_1g_ symmetry in graphene, is intense due to the reduction in size of the in-plane sp^2^ domain; while the *G* peak results from the first order scattering of the E_2g_ phonon of sp^2^ carbon hybridization^33^. In marked contrast to the spectrum of milled graphite (I_D_/I_G_ = ~1.1), the spectrum of nano-NaBH_4_@GNs exhibited a decrease in the relative intensity ratio (I_D_/I_G_ = ~0.24) between the *D* and *G* modes; this ratio is close to that for chemically reduced GO using alkali metals in molten halide flux (I_D_/I_G_ = ~0.22)^33^, indicating the increase of the sp^2^-hybridized network with lesser defects and disorder in the nano-NaBH_4_@GNs. More interestingly, another band at ~2660 cm^−1^ was observed, and its intensity was found to increase significantly in nano-NaBH_4_@GNs as compared to that of milled graphite. This band, known as 2*D*, arises from the second order of zone-boundary phonons, for which the intensity is inversely proportional to the defect concentration. Thus, the presence of 2*D* peak is evidence to support the perfect sp^2^ hybridization GNs network with fewer lattice defects. In addition, the 2*D* band, which is sensitive to the number of graphene layers, exhibits a remarkable enhancement, again providing key information about the exfoliation of graphite into few-layered structure. The large-area Raman mapping in Fig. 2c further shows unobvious color gradations of wine, green and blue across the nano-NaBH_4_@GNs surface that plots the spatial dependence of the *D*, *G* and 2*D* band intensity, strongly indicating a uniform multilayer nature of the exfoliated GNs by MFSP.

X-ray photoelectron spectroscopy (XPS) was employed to identify chemical composition and bonding states of the exfoliated GNs. The C 1s XPS spectrum of nano-NaBH_4_@GNs (Fig. 2d) clearly indicates a degree of oxidation fitted to three components, corresponding to carbon atoms bound in several different states: in-plane sp^2^ carbon (~284.7 eV), CO bound carbon (~285.6 eV) and carboxylate carbon (OCO, ~289.4 eV), which are consistent with the previous reports^32^,^33^. The intensities of carbon-oxygen binding energies are greatly decreased as compared to that of carbon-carbon binding energy. This indicates the partial removal of oxygen containing functional groups in nano-NaBH_4_@GNs, which is most likely due to the presence and its reduction effect of NaBH_4_ (as evidence from the Na 1s XPS spectrum). The AFM along with HRTEM images in Supplementary Figs S5 and S6 further demonstrate that the graphite was exfoliated into multilayered nanosheets (thickness of sheet is 2~3 nm). In principle, this 2D layered structure facilitates the homogenous anchoring of NaBH_4_ nanodots onto the GNs matrix.

**Figure 3:**
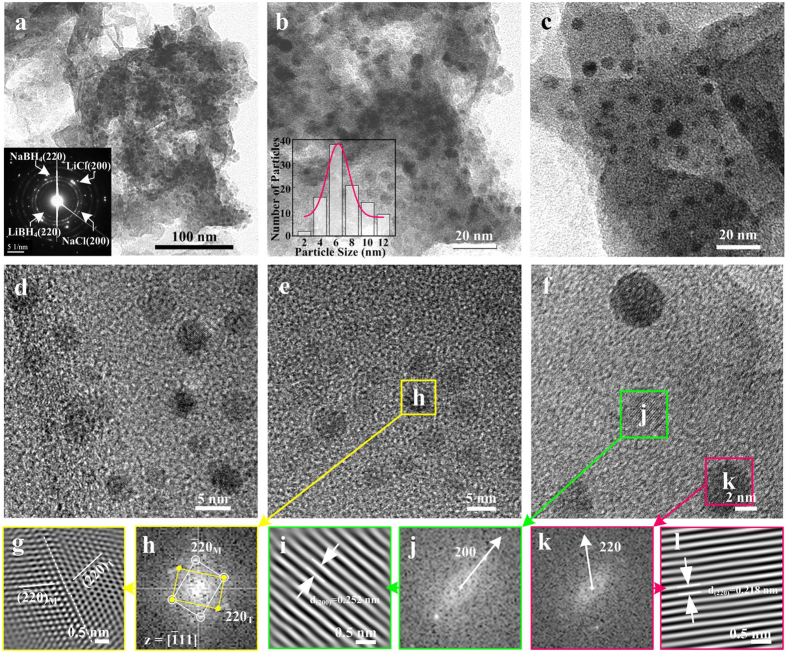
Morphological verification of NaBH_4_ nanodots on GNs. (**a**) TEM image for the nano-NaBH_4_@GNs and the corresponding selected-area electron diffraction (SAED) pattern (inset of **a**); (**b,c**) TEM micrographs of nano-NaBH_4_@GNs; and the corresponding histogram for the particle size distribution of nanodots (inset of **b**). The average nanodot diameter is 6.2 ± 0.8 nm; (**d–f**) High-resolution TEM image showing well-dispersed nanodots; (**g,i,l**) atomic lattice image of the square regions in (**e,f**) displaying the distribution of pseudo-twin and single NaBH_4_ as well as LiCl nanocrystals obtained by inverse fast Fourier transform (IFFT); (**h,j,k**) fast Fourier transform (FFT) digital diffractograms of the nanodomains marked with yellow, green and pink squares in (**e,f**), respectively, revealing the presence of lattice fringes with a separation of 0.218 and 0.252 nm, in agreement with the (220) and (200) interplanar *d* spacing of NaBH_4_ and LiCl (JCPDS 78-0544, 0.217 nm; JCPDS 89-3611, 0.254 nm), respectively.

The representative TEM images and the corresponding SAED pattern and size distribution analysis of the nano-NaBH_4_@GNs are displayed in Fig. 3a–f. These images show particles with an approximately spherical morphology and an average diameter of 6.2 ± 0.8 nm; TEM analysis of more than 100 NCs from more than six different syntheses indicate that the particles were well distributed throughout the GNs, with no evidence of agglomeration. The clear rings composed of discrete spots in the SAED pattern demonstrate the existence of NaBH_4_, LiCl and residual NaCl phases, which agrees well with the obtained XRD results (Fig. 2a). The corresponding C and O maps followed the morphology of the aggregates as marked by the white square, and the relatively scattered Na morphology coincides well with the dense C and O maps (Supplementary Fig. S7). Figure 3g–l reveal the atomic-resolution images of the NaBH_4_ and LiCl basal planes with open edges, whereby NaBH_4_ unexpectedly existed as some single and some twin crystallites in each nanodot. These features demonstrate that MFSP resulted in the ultrasmall NaBH_4_ nanodots uniformly self-assembled onto GNs.

### Thermodynamic and kinetic destabilizations of nano-NaBH_4_@GNs composites

**Figure 4:**
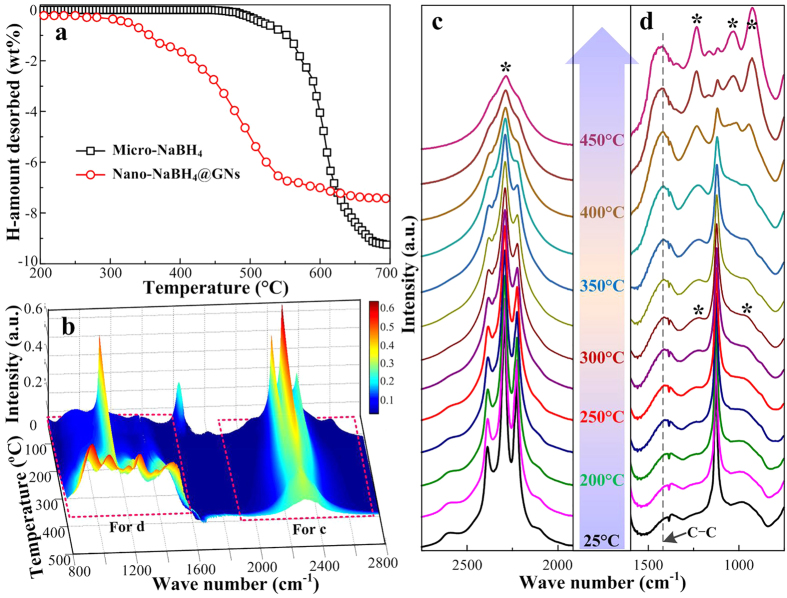
Temperature-programed hydrogen desorption monitoring. (**a**) TGA curves for the micro-NaBH_4_ and nano-NaBH_4_@GNs measured at 5 °C min^−1^; (**b**) 3D evolution profile of the *in situ* FTIR spectrum for the nano-NaBH_4_@GNs upon heating to target temperatures at 5 °C min^−1^; (**c,d**) selected high-resolution FTIR spectra of the nano-NaBH_4_@GNs from (**b**). The peaks marked by * in **d** are attributed to the characteristic absorption frequencies of boron-containing species. For comparison, the hydrogen capacity of nano-NaBH_4_@GNs was normalized to the weight of NaBH_4_ without the disturbance of both residuals and GNs.

Figure 4a compares the decomposition behaviors of nano-NaBH_4_@GNs and commercial NaBH_4_ (~40 mesh, denoted as micro-NaBH_4_) by thermogravimetric analysis (TGA). The nano-NaBH_4_@GNs exhibited a remarkable decrease in both the onset and completion temperatures for a weight loss of ~7.0 wt% at approximately 250 and 550 °C, respectively, in marked contrast to the ~9.1 wt% mass loss from about 490 °C to 700 °C for micro-NaBH_4_. To verify that the weight loss originated from the borohydride desorption, the bonding evolution of nano-NaBH_4_@GNs upon heating was determined by *in situ* Fourier transform infrared spectroscopy (FTIR). Figure 4b–d display the temperature-resolved FTIR spectra for the nano-NaBH_4_@GNs. In the spectra collected at room temperature (~25 °C), four distinct, intense peaks are observed at ~2385, 2292, 2225 and 1126 cm^−1^, corresponding to the characteristic B–H stretching (first three of the four peaks) and bending (last one of the four peaks) vibration frequencies for the [BH_4_]^−^ units, respectively^19^. As the temperature was increased to 250 °C, the relative intensity of the peaks for the [BH_4_]^−^ units started to decrease in relation to the intensity of the initial ones, in good agreement with the TGA results (Fig. 4a). When the temperature was increased to 300 °C, with the exception of the weaker peaks for the [BH_4_]^−^ units, two additional peaks at ~1232 and 926 cm^−1^ appeared and were identified as the frequencies for the boron-containing species^34^,^35^, implying the direct decomposition of borohydride into its components. Notably, the characteristic [B_12_H_12_]^2−^ peak at ~2248 cm^−1^ was not observed in the initial process, which is further evidence supporting the altered dehydrogenation pathway of ultrafine NaBH_4_ NCs in this work. This result is different from the previous results indicating that *M*_2_B_12_H_12_ is one of the desorption intermediates formed before the final thermal decomposition^19^. Throughout the process from 25 to 450 °C, the three sharp peaks in Fig. 4c gradually merged into a single broad peak, whereas a single primary peak in Fig. 4d separated into three new peaks at ~1232, 1037 and 926 cm^−1 ^34,35; all these peaks are ascribed to the desorption products for the borohydride, i.e., the polymorphic boron and/or boron-containing species. This enhanced desorption of NaBH_4_ is clearly attributed to the dramatic nanosize effect rather than the effect of ball-milling and/or catalysis by the additions of graphite or LiCl, as supported by the comparison of the dehydrogenation and FTIR features in Supplementary Figs S8 and S9.

**Figure 5:**
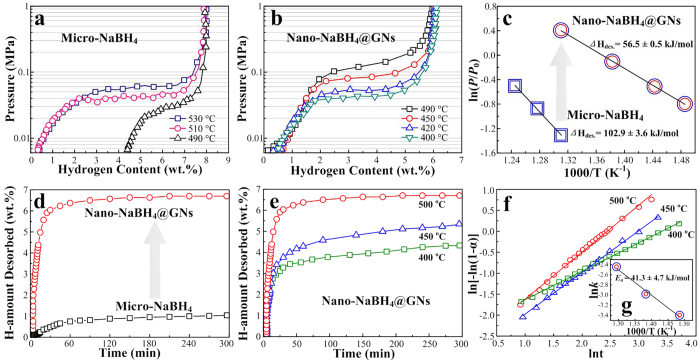
Thermodynamic and kinetic destabilizations in desorption. (**a,b**) *P−C* desorption isotherms measured at different temperatures; (**c**) van’t Hoff plots at the equilibrium pressures were obtained from the midpoints of the *P−C* desorption isotherms in (**a, b**); (**d**) isothermal hydrogen desorption curves measured at 500 °C; (**e**) isothermal hydrogen desorption curves for the nano-NaBH_4_@GNs composites measured at various temperatures; (**f**) the Johnson-Mehl-Avrami (JMA) model plots of 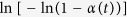 versus ln*t* and Arrhenius plot (inset of **g**) for the isothermal dehydrogenation of the nano-NaBH_4_@GNs composites. For comparison, the hydrogen capacity of nano-NaBH_4_@GNs was normalized to the weight of NaBH_4_ without the disturbance of both residuals and GNs.

To reveal the superior hydrogen storage properties of the nano-NaBH_4_@GNs, we conducted a series of de-/re-hydrogenation measurements. As evident in Fig. 5, two positive aspects are observed: (i) the thermodynamics of the desorption was destabilized (Fig. 5a–c). For example, the plateau pressure of dehydrogenation at 490 °C increased from approximately 0.025 MPa for micro-NaBH_4_ to about 0.18 MPa for the nano-NaBH_4_@GNs, and the corresponding decomposition reaction enthalpy *∆H* decreased from 102.9 ± 3.6 kJ mol^−1^ of H_2_ for micro-NaBH_4_ (similar to the value of 108 ± 3 kJ mol^−1^ of H_2_ reported by Martelli^36^) to 56.5 ± 0.5 kJ mol^−1^ of H_2_ for nano-NaBH_4_@GNs; and (ii) the desorption kinetics was also distinctly enhanced (Fig. 5d–g). For example, ~6.8 wt% of hydrogen was released in 120 min from the nano-NaBH_4_@GNs, whereas only ~1 wt% was released from micro-NaBH_4_ at 500 °C within 300 min. More importantly, the nano-NaBH_4_@GNs sample still showed favorable kinetics at an even lower temperature; e.g., it released ~5.2 wt% and 4.1 wt% H_2_ in 300 min at 450 and 400 °C, respectively, whereas the micro-NaBH_4_ did not release any hydrogen at 400 °C (results not shown).

We further characterized this improved dehydrogenation kinetics by calculating the activation energy *E*_a_ (details in the supplemental materials), where the *E*_a_ was 41.3 ± 4.7 kJ mol^−1^ for the nano-NaBH_4_@GNs, which is dramatically reduced by ~80% relative to the reported *E*_a_ of ~220 kJ mol^−1^ for the micro-NaBH_4_ even doped with some fluorides^37^. To the best of our knowledge, such a low *E*_a_ for hydrogen desorption from NaBH_4_ has not been previously reported. These results clearly indicate that improvements in both the thermodynamics and kinetics are indeed achieved in the nano-NaBH_4_@GNs composites.

### Enhanced reversibility of nano-NaBH_4_@GNs composites

**Figure 6:**
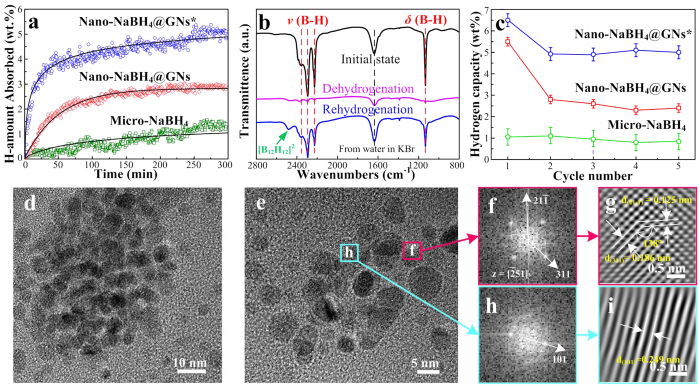
Stable capacity and nanostructure in reversible hydrogen absorption. (**a**) Re-hydrogenation curves measured at 450 °C under 6 MPa hydrogen pressure; (**b**) selected-region FTIR spectra for the nano-NaBH_4_@GNs^*^ composites in the initial, dehydrogenated and re-hydrogenated states. The B–H stretching vibration bands for [BH_4_]^−^ units located at about 2385, 2292 and 2225 nm^−1^. Note that peak at about 2480 nm^−1^ is attributed to the stretching vibration band for [B_12_H_12_]^2−^ units, indicating the intermediate for the reversible reaction; (**c**) the reversible hydrogen storage capacities as a function of the number of cycles; (**d,e**) HRTEM images for the nano-NaBH_4_@GNs^*^ after five cycles; (**f**) FFT digital diffractogram and (**g**) IFFT-TEM atomic image of the NaBH_4_ NCs inside the pink box in (**e**), revealing the presence of lattice fringes with a separation of 0.186 and 0.125 nm, in agreement with the (311) and (21-1) interplanar *d* spacing of NaBH_4_ (JCPDS 04-0770, 0.185 and 0.125 nm), respectively; (**h**) FFT digital diffractogram and (**i**) IFFT-TEM atomic image of the MgH_2_ NCs inside the cyan box in (**e**), revealing the presence of lattice fringes with a separation of 0.249 nm, in agreement with the (101) interplanar *d* spacing of MgH_2_ (JCPDS 74-0934, 0.251 nm). For comparison, the hydrogen capacity of nano-NaBH_4_@GNs and nano-NaBH_4_@GNs* were normalized to the weight of NaBH_4_ without the disturbance of both residuals and GNs.

Figure 6a shows the remarkable improvement in the reversibility of nano-NaBH_4_@GNs compared with that of micro-NaBH_4_. The micro-NaBH_4_ exhibits an absorption of ~1 wt%, whereas a much higher reversible capacity of approximately 3 wt% was achieved for the nano-NaBH_4_@GNs. However, this reversible capacity is obviously still lower than the initial desorption value of ~7 wt% in Fig. 4a. To reveal the intrinsic degradation mechanisms with the goal of further improvement, we investigated the phase evolution of the nano-NaBH_4_@GNs upon de-/re-hydrogenation in detail. As shown in Supplemental Fig. S10, the NaBH_4_ decomposed into Na and amorphous B, and the Na then reacted with LiCl to form NaCl and Li, corresponding to the presence of LiH even under an atmosphere with ultratrace levels of desorbed H_2_. The stable LiH was difficult to reversibly decompose under the present conditions, thus resulting in the loss of elemental Na for the effective recovery.

To overcome the limited reversibility, a chemical alloying strategy was adopted whereby additional MgH_2_ with a 1:2 molar ratio of Mg:B atoms was introduced (i.e., nano-NaBH_4_@GNs^*^). As expected, the nano-NaBH_4_@GNs* absorbed ~5 wt% of H_2_ under the same conditions of 450 °C and 6 MPa (Fig. 6a). Further combining the FTIR and XRD results (Fig. 6b and Supplemental Fig. S11), we observed that the introduced Mg reacted with Li to form Li_3_Mg_17_ instead of stable LiH. Upon hydrogenation, the Li_3_Mg_17_ released active Li for to participate in the reversibility, thus achieving the recovery of NaBH_4_ and MgH_2_ (as demonstrated below). Note that the occurrence of [B_12_H_12_]^2−^ bonding frequencies at 2480 cm^−1^ in Fig. 6b further suggests unlike the dehydrogenation (Fig. 4c), the re-hydrogenation involved the Na_2_B_12_H_12_ intermediate^38^,^39^. Figure 6c shows a higher H_2_ storage capacity of ~5 wt% with ~77% retention of the initial value after five cycles for nano-NaBH_4_@GNs*. By contrast, the nano-NaBH_4_@GNs and micro-NaBH_4_ exhibited a lower capacity of ~2.5 and ~1 wt%, respectively, even with higher retentions. Even more significantly, these ultrafine dot nanostructures were maintained perfectly without obvious growth or agglomeration (Fig. 6d,e). The FFT digital diffractogram and corresponding atomic images in Fig. 6f–i indeed demonstrate the recovery of NaBH_4_ and MgH_2_, in agreement with the phase and bonding results in Fig. 6b and Supplemental Fig. S11. These results clearly indicate that the nano-NaBH_4_@GNs* exhibit not only a high cyclic capacity but also stable nanostructures.

## Discussion

In this study, the formation process involved in Route II mimics the process used in a ‘dye inject printer’ whereby the borohydride nanocrystals acting as a ‘dye’ is uniformly printed on the exfoliated few-layered graphitic structure as a ‘paper board’ and, therefore, which is the proof-of-concept of ‘mechanical-force-driven self-printing (MFSP)’ proposed by us for the synthesis of metal borohydrides at the ultrasmall nanoscale. During the MFSP process, the realization of monodisperse NaBH_4_ nanodots self-printed on the *in situ* prepared GNs should be ascribed to the synergic effect of the following actions: (i) the mechanical shear force directly crushes the agglomerates and facilitates the intercalation of various functional groups into the broken edges of the graphite in the presence of the appropriate chemicals^40^; (ii) similar to the interaction between NH_3_BH_3_ and the *sp*^2^-carbon lattice network of graphite, the borohydride molecules can be readily adsorbed/spread onto the surface of graphite (i.e., being convenient for the writing of MFSP) due to the electrostatic interaction under ball-milling conditions^30^,^41^,^42^. This adsorption/spreading of borohydride may weaken the van der Waals interactions between the graphite interlayers, allowing graphitic sheets to be easily exfoliated as ‘paper board’ for writing, as demonstrated by the presence of some slightly platelet-like structures in the milled graphite with commercial micro-sized borohydrides; (iii) smaller NaBH_4_ NCs results in milder intercalation into the graphite interlayers and easier disruption of the van der Waals-like forces, thus leading to effective exfoliation into ultrathin layered graphite; this effect further explains why the *in situ* formed NaBH_4_ NCs are more favorable than commercial micro-sized NaBH_4_ for the exfoliation of graphite into ultrathin layered structures; and (iv) taking a role in controlling the structure^6^,^43^, the high-surface-area GNs serve as a novel ‘paper board’ in mediating the nucleation and growth of NaBH_4_ nanodots as ‘dye’ that not only provide more active sites and lower energy barriers for the adsorption, i.e., being easier to write; but also restrict the growth of these nuclei by the interface interactions, i.e., shape-control in printing.

Furthermore, the substantial improvements in the de-/re-hydrogenation behaviors observed in Figs 4, 5, 6 can be understood from five aspects: (i) the nanosized NaBH_4_ is thermodynamically less stable because of its higher specific surface area and abundant grain boundaries than those of the bulk material^44^,^45^; (ii) the bond distances in the surfaces of the nanostructure are greater than those in the bulk material and the corresponding H-site energies make the removal of hydrogen from the nanostructure surface easier than that from the bulk surface^18^; (iii) at the nanoscale level, the inter-diffusion of constituents during de-/re-hydrogenation process occurs over a short-range, which differs from the long-range transfer in the bulk NaBH_4_ that is the common rate-limiting step for de-/re-hydrogenation^29^; (iv) the 2D GNs not only serve as a nanosupport for dispersion that prevents the aggregation and growth but also facilitate heat transfer because of their excellent thermal conductivity^44^; and (v) positive effect of the chlorides byproducts. For example, the LiCl reacted with Na products to form NaCl and Li during dehydrogenation process, which not only results in promotion of NaBH_4_ decomposing into Na and amorphous B but also alleviates the migration and loss of liquid Na for the effective recovery. While for the hydrogenation, the NaCl was found to be reacting with Li (from Li_3_Mg_17_ decomposition) for *in situ* producing the high active Na species that closes to the resulting B species on graphite nanosheets, which is the key factor for the improved regeneration of NaBH_4_.

In summary, ultrasmall (~6 nm) NaBH_4_ nanodots with excellent dispersibility have been successfully constructed on freshly prepared graphitic nanosheets by a mechanical-force-driven self-printing method. The keys to achieving printing of NaBH_4_ nanodots on newly exfoliated GNs from graphite are the coupling effects of a moderate mechanical shear force, efficient intercalation of borohydride, the structure-directing role of GNs, and the strong interaction of borohydride with GNs. The obtained nano-NaBH_4_@GNs exhibit mild desorption starting at ~250 °C, rapid kinetics with a record low *E*_a_ value of 41.3 ± 4.7 kJ mol^−1^ and a better cyclic de-/re-hydrogenation with exceptional nanostructure stability even after five cycles. In addition, we believe that this study may introduce an effective, facile protocol for fabricating ultrasmall and nearly monodispersed zero-dimensional complex hydrides with high loading on two-dimensional nanosupports for potential applications, including hydrogen storage, solid-state electrolyte, catalysis, etc.

## Methods

All operations were carried out under inert atmosphere in an argon-filled glove box (<1 ppm O_2_ and H_2_O, MBRAUN, Germany). All chemicals were purchased from Sigma-Aldrich (United States) and used without further purification.

### Synthesis of nano-NaBH_4_@GNs

In a typical procedure, 0.5 g of raw materials consisting of LiBH_4_ (95% purity, ~40 mesh) and anhydrous NaCl (99.9% purity, ~40 mesh) mixed in a 1:1 molar ratio was mechanically milled with graphite (99.9% purity, ~300 mesh) in a mass ratio of 10:1 for 10 h at ambient temperature under an argon atmosphere using a planetary mill at a speed of 400 rpm with a 60:1 ball-to-powder ratio (i.e., Route II, nano-NaBH_4_@GNs). For comparison, the same procedure was carried out on a LiBH_4_ and NaCl mixture in a 1:1 molar ratio (i.e., Route I) and on bare graphite (i.e., Route III). Moreover, the mixture of as-prepared nano-NaBH_4_@GNs and MgH_2_ (98% purity, ~100 mesh) with a 2:1 molar ratio of B:Mg atoms was milled for 2 h at 200 rpm to obtain nano-NaBH_4_@GNs*.

### Characterizations

HRTEM and scanning electron microscopy (SEM) observations were carried out on an FEI Tecnai G2 F30 transmission electron microscope equipped with an energy dispersive X-ray spectroscopic analyzer (EDX) and on a Shimadzu SUPERSCAN SSX-550, respectively. For the HRTEM observations, the samples were directly spread onto a holey carbon film supported on a copper grid to avoid the disturbing effect of solvents on the borohydride nanostructures. X-ray diffraction (XRD) was carried out on a Rigaku D/max 2400 diffractometer equipped with a Cu Kα radiation source operated at 50 kV and 30 mA. XRD samples were exclusively handled in an Ar-filled glove box, and the surfaces of the samples were covered with Scotch tape to prevent any possible reaction with water or oxygen during measurement. Raman spectroscopy was carried out on a HORIBA Jobin Yvon LabRAM HR800 (Horiba Scientific) confocal Raman microscope excited by a 442 nm helium–cadmium (He–Cd) blue laser. The Raman signal was collected through a confocal pinhole of 250 µm diameter by using a 0.90 NA dry objective of 100 × magnification (OLYMPUS, Japan). AFM was performed on a Veeco NanoScope IIIA AFM instrument in tapping mode. Raman mapping and AFM samples were prepared by directly dispersing nano-NaBH_4_@GNs powders on 4 × 4 mm silicon substrates. X-ray photoelectron spectroscopy (XPS) was collected using a Kratos Axis Ultra X-ray photoelectron spectrometer, equipped with an aluminum/magnesium dual anode and a monochromated aluminum Kα X-ray sources. The thermal desorption behaviors of the as-prepared samples were studied by TGA on a Netzsch STA 409 PC; the sample was heating at 5 °C min^−1^ under a flowing Ar atmosphere. All samples were transferred into the equipment by means of a device that maintained an Ar overpressure during the transfer process. The *in situ* FTIR measurements were carried out on a Nicolet iS50 with a sealed reaction cell to examine the evolution of the chemical bonds under an Ar atmosphere (99.999% purity) at temperatures ranging from room temperature to 450 °C at a ramp rate of 5 °C min^−1^. The specimen for *in situ* FTIR measurements was a mixture of nano-NaBH_4_@GNs and dried KBr in a weight ratio of 1:100 and was filled into a sample holder in the sealed reaction cell.

### Thermodynamics and kinetics measurements

The desorption thermodynamics and kinetics were measured using an automated Sieverts-type apparatus that allowed for the accurate determination of the amounts of evolved hydrogen. Typically, approximately 0.5 g of each sample was loaded into a stainless-steel autoclave and evacuated. The sample was rapidly heated to the desired temperatures by immersing the autoclave into a silicon oil bath preheated to a given temperature. Hydrogen desorptions at different temperatures were successively performed against a back pressure of 10 Pa.

### Cycling tests

The changes in capacity with multiple hydrogen ab/desorption cycling were also studied by Sieverts-type measurements. A sample of about 0.5 g sealed in the autoclave was rapidly heated to 450 °C and maintained at this temperature for hydrogen release. After hydrogen release was completed, the sample was pressurized with 6 MPa H_2_ for 5 h for absorption. The same hydrogen ab/desorption procedure was repeated several times to examine the cycling performance.

## Additional Information

**How to cite this article**: Li, Y. *et al*. Self-Printing on Graphitic Nanosheets with Metal Borohydride Nanodots for Hydrogen Storage. *Sci. Rep*. **6**, 31144; doi: 10.1038/srep31144 (2016).

## Supplementary Material

Supplementary Information
