# Supplementary material for: Self-Printing on Graphitic Nanosheets with Metal Borohydride Nanodots for Hydrogen Storage
Source: Sci Rep. 2016 Aug 3;6:31144. doi: 10.1038/srep31144 (PMC4971464; doi:10.1038/srep31144)
Supplement: Supplementary Information [file srep31144-s1.pdf]

Supplementary materials

Self-Printing on Graphitic Nanosheets with Metal Borohydride Nanodots for Hydrogen Storage

Yongtao Li[1], Xiaoli Ding[1] & Qingan Zhang[1*]

[1]School of Materials Science and Engineering, Anhui University of Technology, Maanshan 243002, China

Supplementary Note 1:

Activation energy for hydrogen release kinetics determined by JMAK model. The enhanced dehydrogenation kinetics of nano-$NaBH_4$@GNs was characterized by calculating the activation energy E_a for hydrogen release. Here, the E_a for hydrogen release is determined by using JMAK (Johnson-Mehl-Avrami-Kolmogorov) model analysis[1]. Base on the JMAK model, the hydrogen release kinetics can be expressed by the following equation:

$$\ln[-\ln(1-\alpha)] = \eta \ln k + \eta \ln t \qquad \text{(S1)}$$

where k is the rate constant; α is the reaction fraction transforming from 0 to 1, corresponding to beginning and completion of the reaction; η is the Avrami exponent of the reaction order and t is the time. For the experimental data of the samples, the linearity of plotting ln[-ln(1-α)] against ln(t) is achieved for each curve at different temperatures, as shown in **Fig. 5f**. After calculating the rate constant k, the activation energy E_a for the hydrogen desorption process can be evaluated from the Arrhenius equation[2]:

$$k = k_0 e^{-E_a/RT} \qquad \text{(S2)}$$

where k_0 is a temperature-independent coefficient, R is the gas constant (8.314 J mol•K^{-1}) and T is the absolute temperature. The linearity plots of ln(k) against 1000/T for hydrogen desorption of nano-$NaBH_4$@GNs are shown in **Fig. 5g**. From the slopes ($-E_a/R$) of the straight lines, the E_a is 41.3 ± 4.7 kJ mol^{-1} for the nano-$NaBH_4$@GNs which is dramatically reduced by ~80% relative to that of ~220 kJ mol^{-1} for the micro-$NaBH_4$ even doped with some fluorides[3]. This result clearly indicates that the dehydrogenation kinetics was indeed enhanced in the nano-$NaBH_4$@GNs composites.

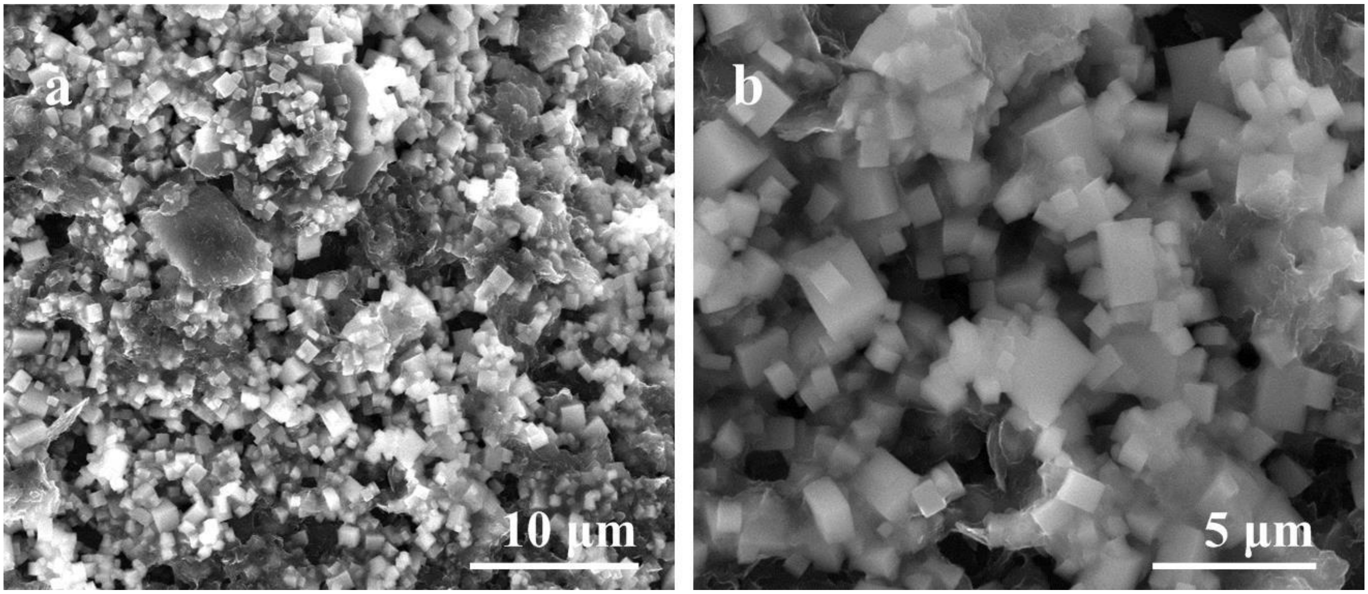

Supplementary Figure S1 | Characterizations for the starting mixture: SEM images of the starting mixture of $LiBH_4$ and NaCl before ball-milling.

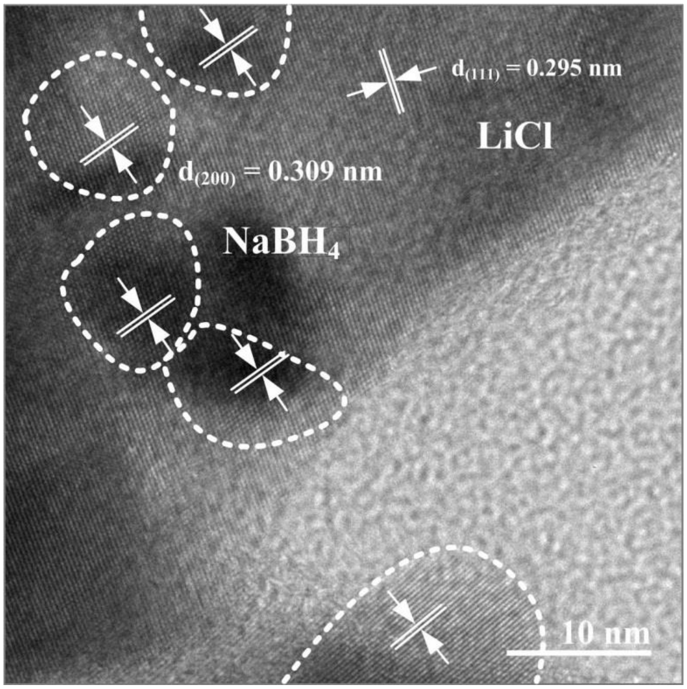

Supplementary Figure S2 | Characterizations for the resulting products of Route I: HRTEM image of the resulting products of $LiBH_4$ + NaCl after ball-milling 10 h.

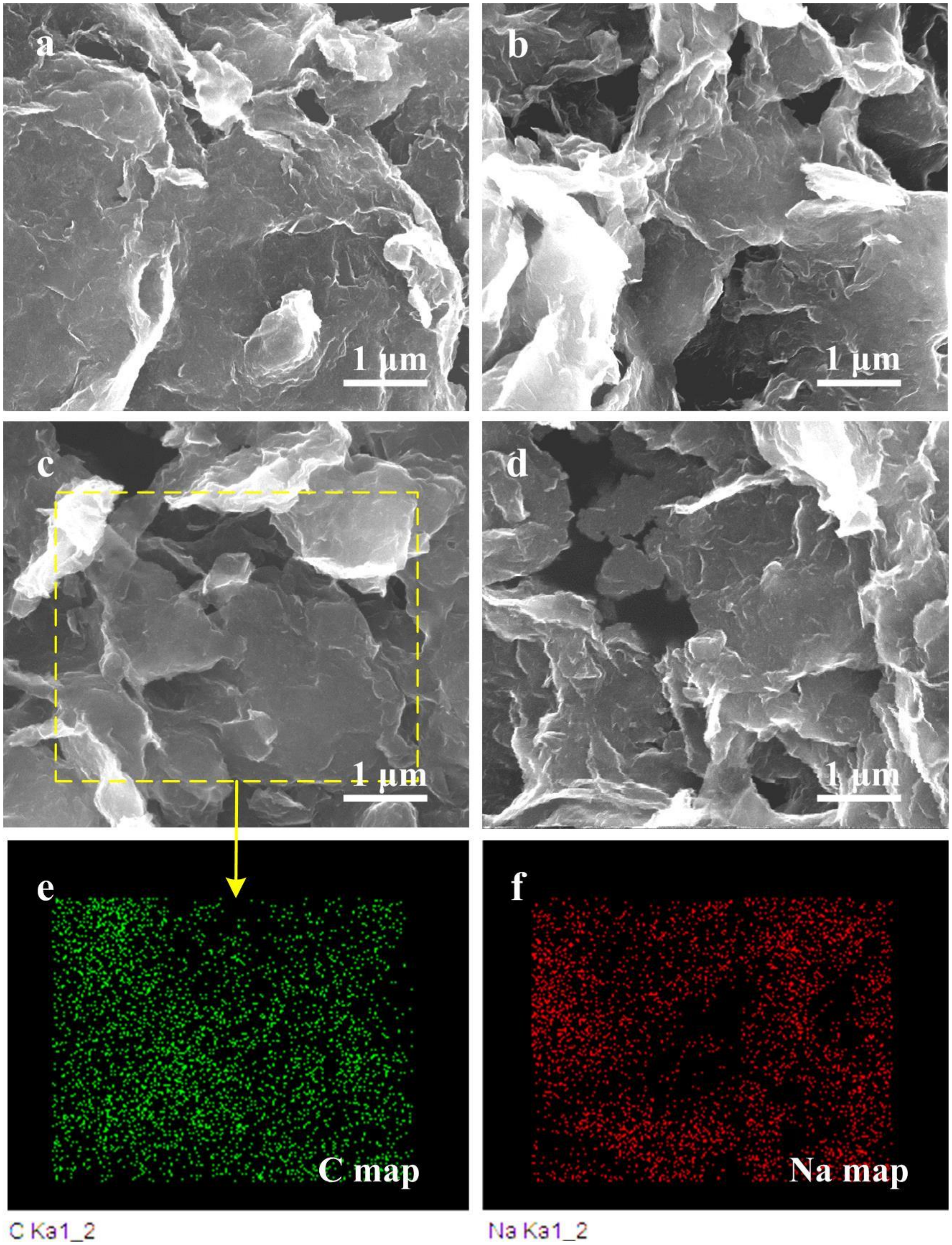

Supplementary Figure S3 | Characterizations for the resulting products of Route II: SEM images **(a–d)** and EDX mapping of carbon (**e**) and sodium (**f**) for the resulting products of graphite + $LiBH_4$ + NaCl after ball-milling 10 h.

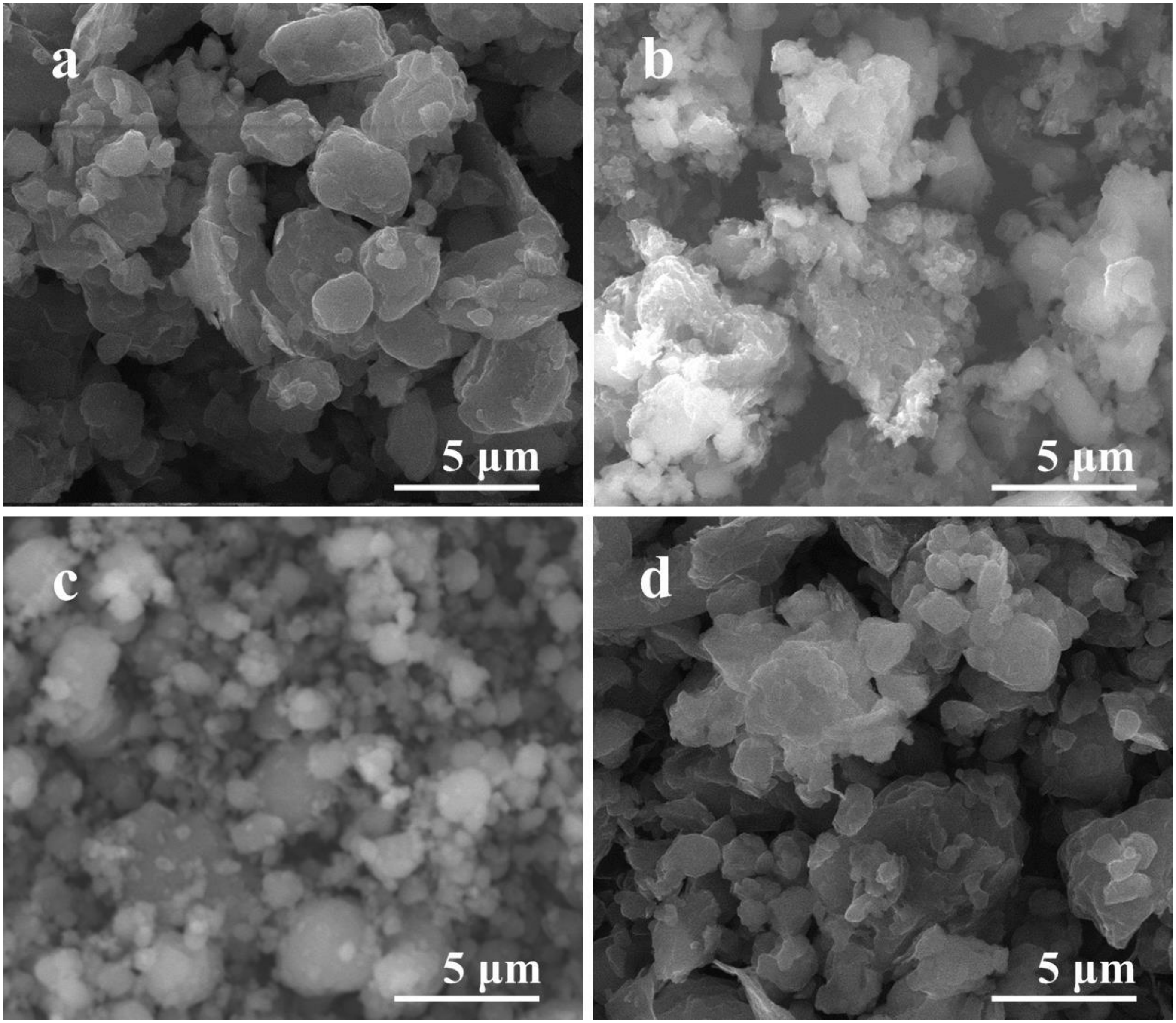

Supplementary Figure S4 | Morphological comparison of the ball-milled products: **(a)** SEM image for graphite + $LiBH_4$, **(b)** SEM image for graphite + NaCl, **(c)** SEM image for graphite + LiCl and **(d)** SEM image for graphite + $NaBH_4$.

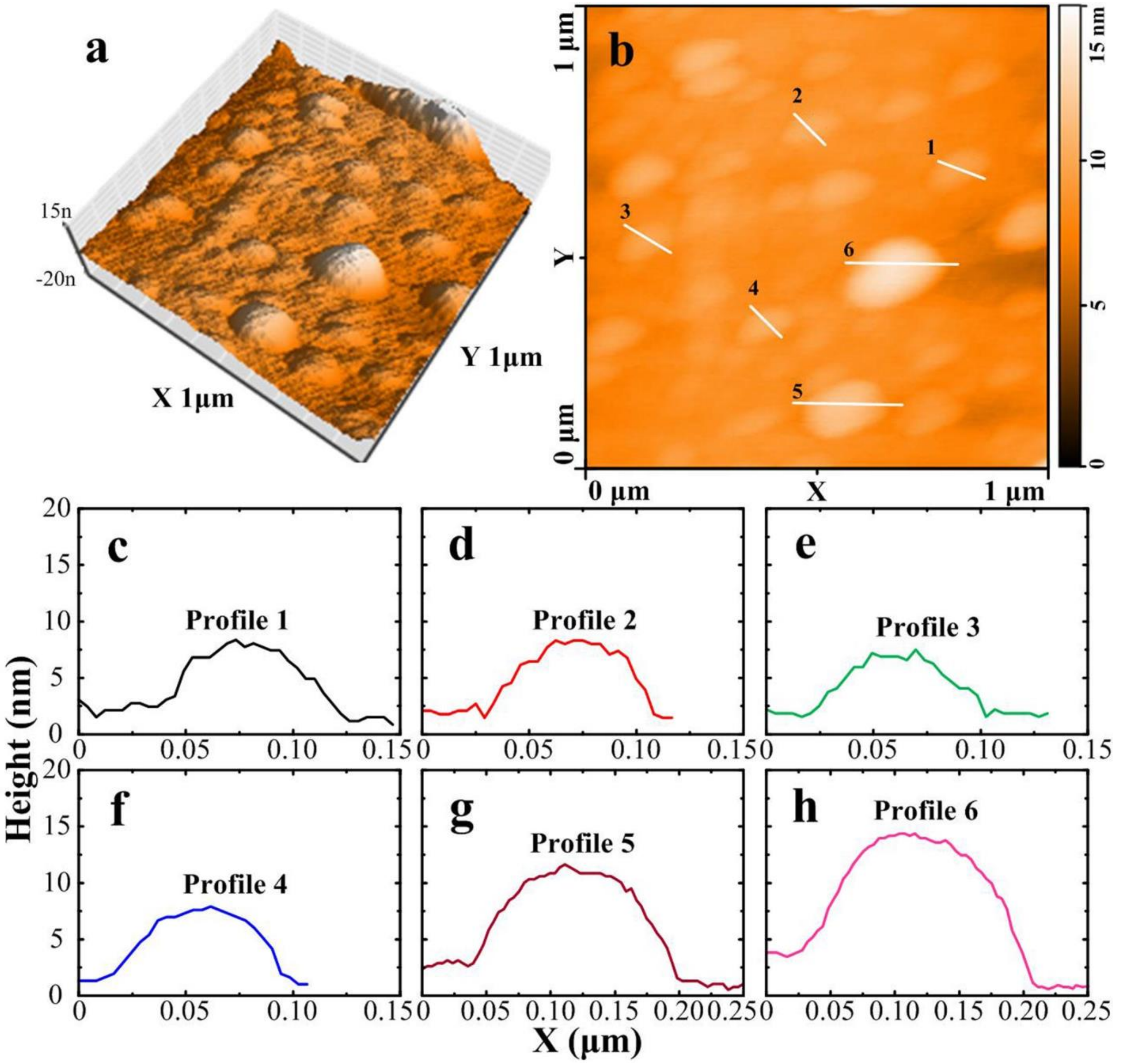

Supplementary Figure S5 | Characterizations for the exfoliated graphitic nanosheets: (a) Three-dimensional (3D) and **(b)** topographic AFM images of the nano-$NaBH_4$@GNs; (**c–h**) Height profiles of the exfoliated multilayered graphitic nanosheets with printed $NaBH_4$ nanodots. The profiles of (**c–h**) correspond to the cross sections of the numbered lines 1, 2, 3, 4, 5 and 6 in Fig. S5b, respectively, showing that the average thickness of GNs plus $NaBH_4$ nanodots is deemed to be about 9.1 nm.

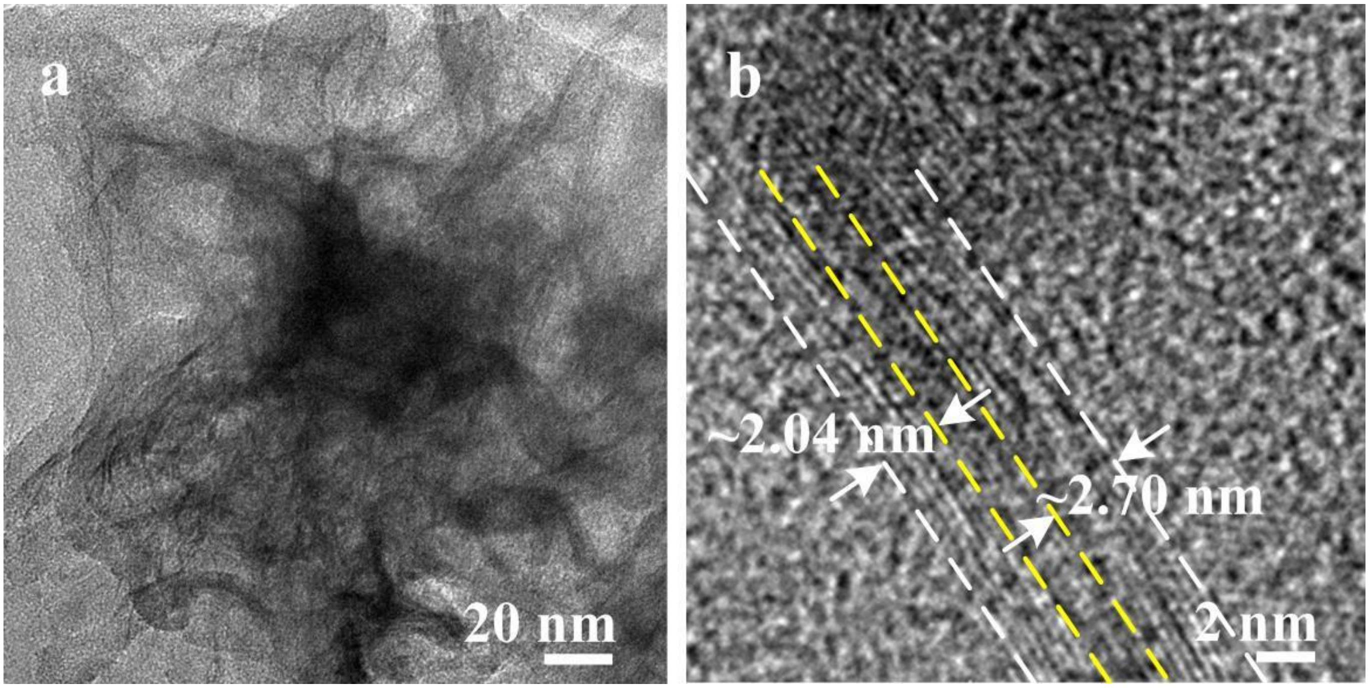

Supplementary Figure S6 | Characterizations for the exfoliated graphitic nanosheets after THF washing: (a) TEM image for the graphitic nanosheets exfoliated from the graphite in Route II after washing by THF; and **(b)** HRTEM image on the walled-edges of the multilayered GNs exfoliated from the graphite in Route II.

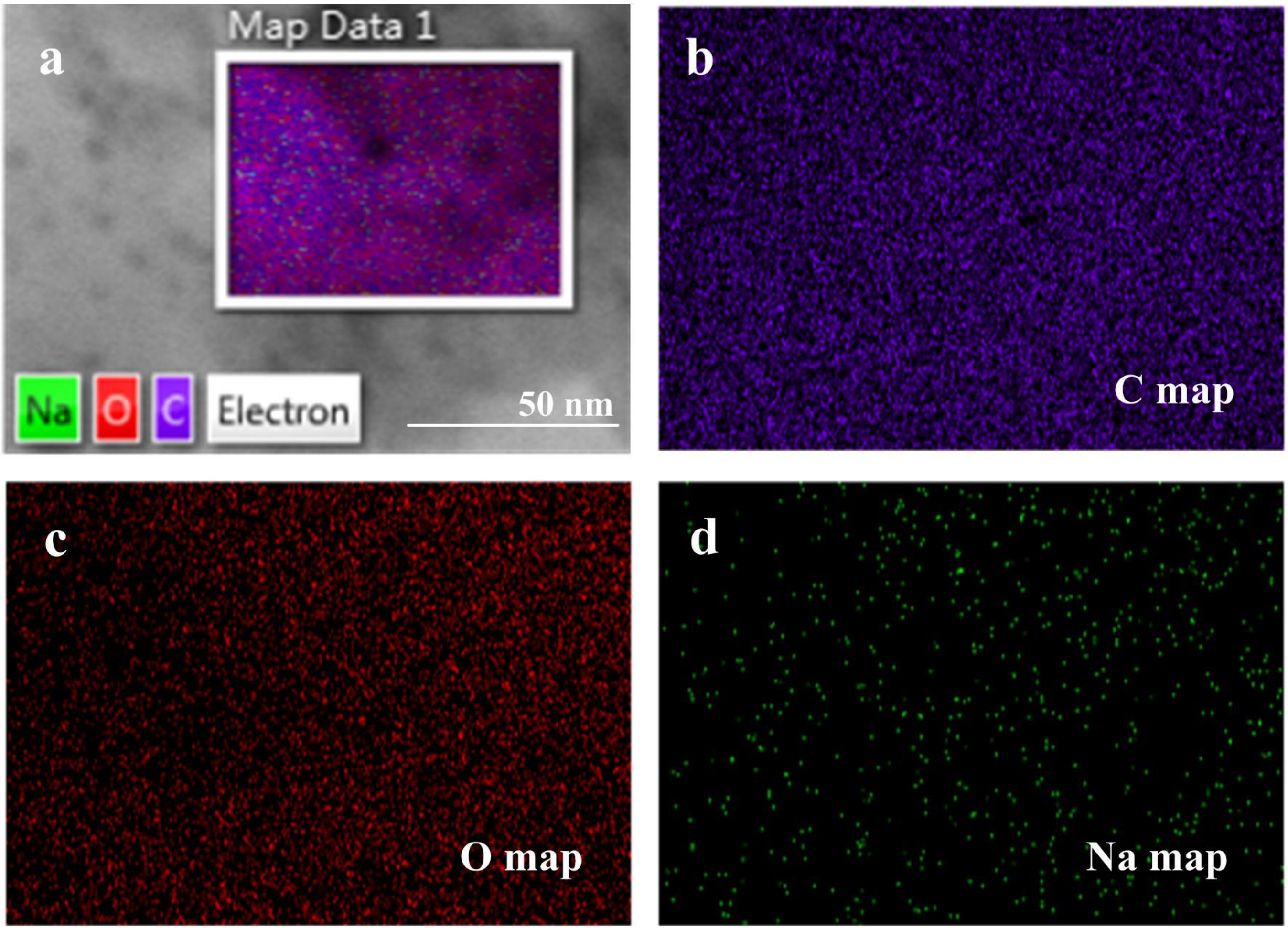

Supplementary Figure S7 | Analysis on chemical composition distribution of the nano-$NaBH_4$@GNs.

(**a**) TEM DF image and selected region for mapping; (**b**) Mapping of carbon (C); (**c**) Mapping of Oxygen (O); **(d)** Mapping of Sodium (Na).

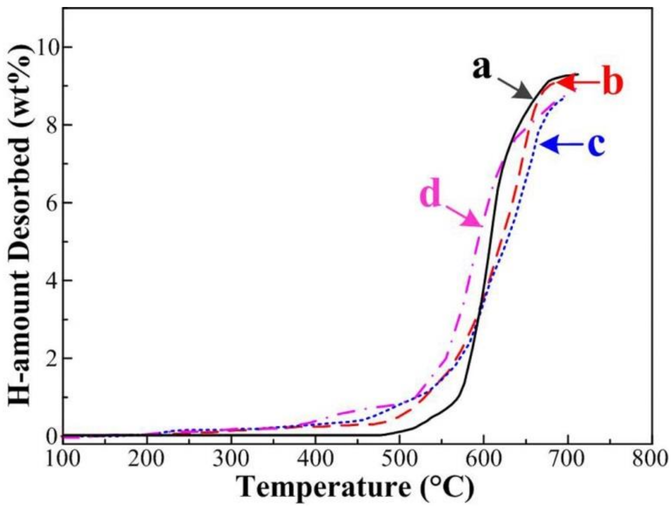

Supplementary Figure S8 | Comparison in dehydrogenation properties: **(a)** $NaBH_4$ (as-purchased), **(b)** $NaBH_4$ (milled 10h), **(c)** $NaBH_4$+graphite (milled 10h) and **(d)** $NaBH_4$+LiCl (milled 10). For comparison, all of capacities were normalized to be the initial weight of $NaBH_4$.

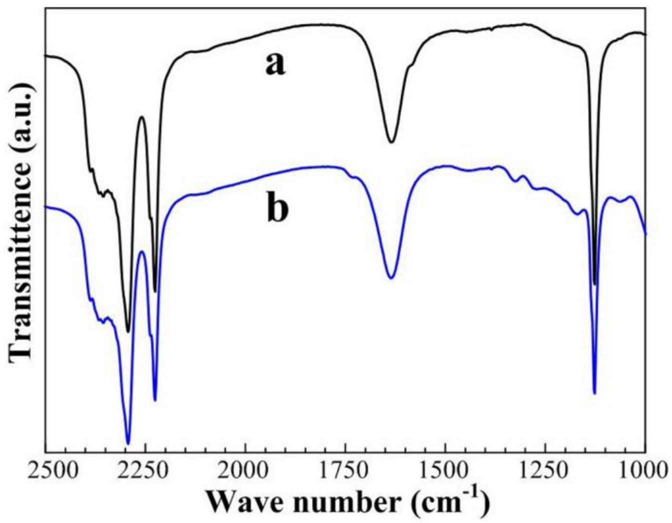

Supplementary Figure S9 | Structural comparison of the milled mixtures: **(a)** FTIR spectrum for $LiBH_4$+NaCl (i.e., Route I) and **(b)** FTIR spectrum for $LiBH_4$+NaCl+graphite (i.e., Route II).

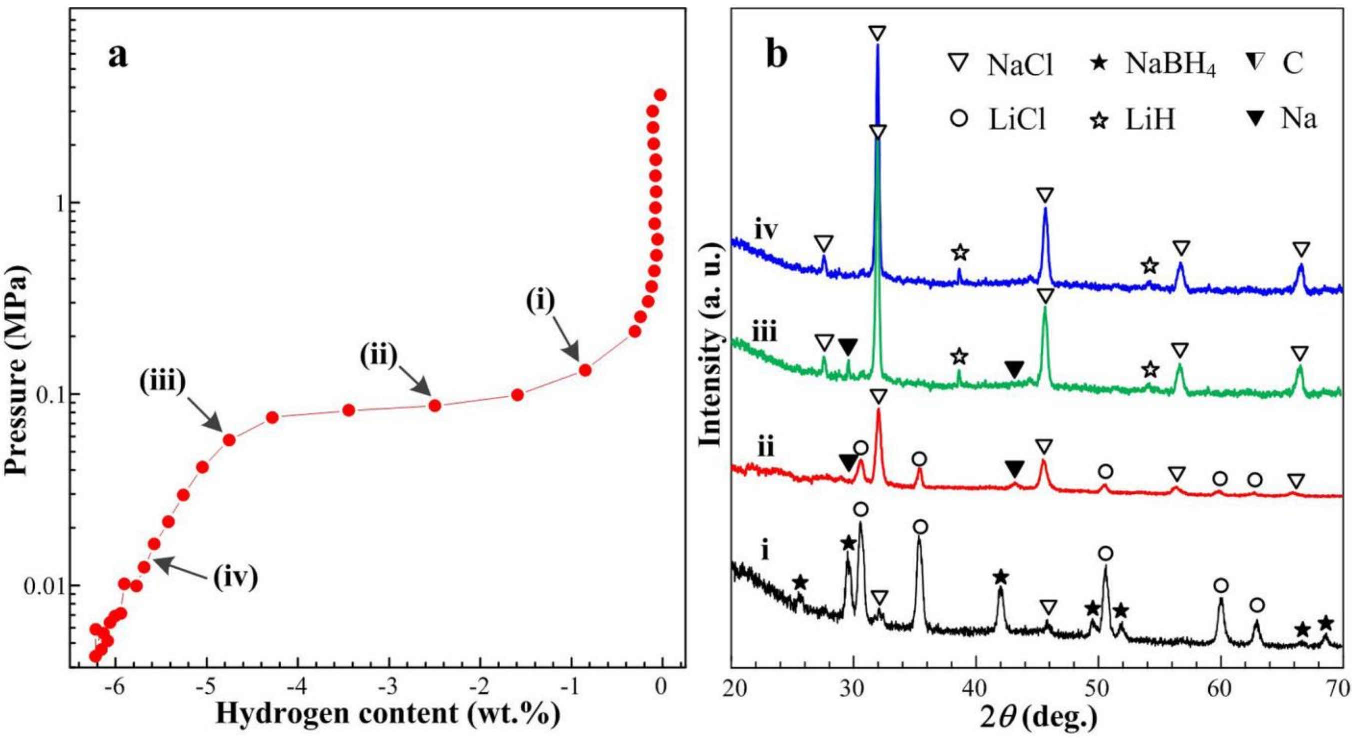

Supplementary Figure S10 | Dehydrogenation process for the nano-$NaBH_4$@GNs: (a) PCT curve for desorption at 450 ℃ and **(b)** *Ex situ* XRD patterns for the selected points from (**a**).

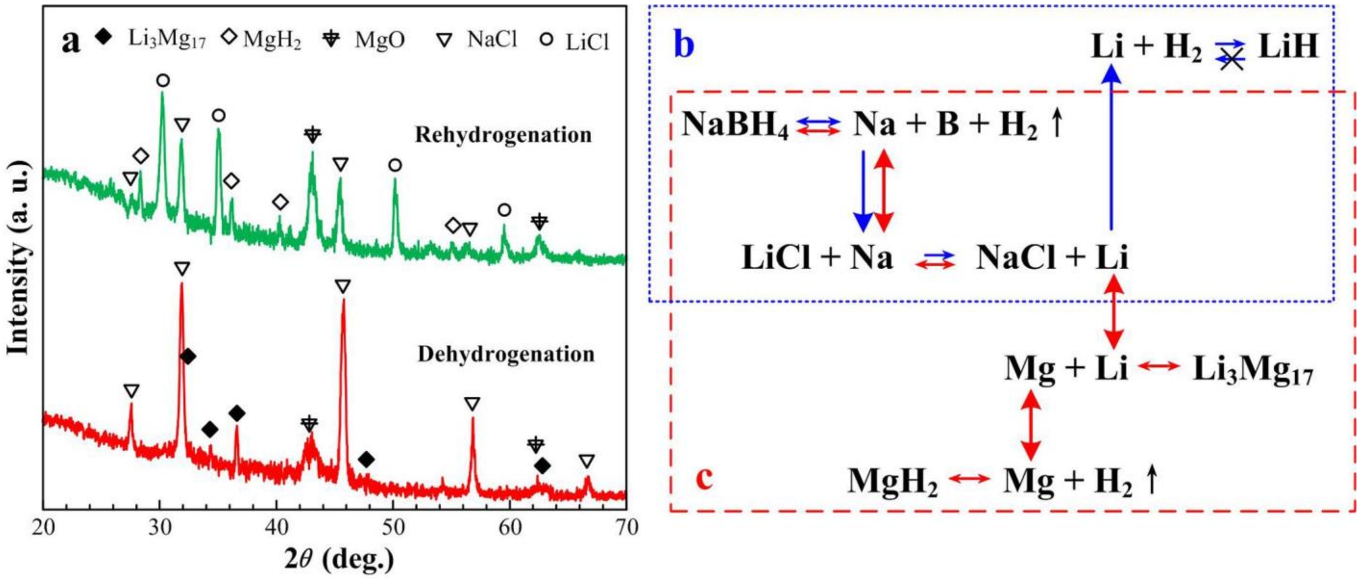

Supplementary Figure S11 | Identification of de-/re-hydrogenation pathways: (**a**) XRD patterns for the de-/re-hydrogenation products of nano-$NaBH_4$@GNs* (i.e., nano-$NaBH_4$@GNs plus MgH_2 with a molar ratio B/Mg of 2:1); and schematic of de-/re-hydrogenation pathways for (**b**) nano-$NaBH_4$@GNs (according to the blue arrows) and (**c**) nano-$NaBH_4$@GNs* (according to the red arrows).

Supplementary References

1. Li, Y. et al. In situ embedding of Mg_2NiH_4 and YH_3 nanoparticles into bimetallic hydride $NaMgH_3$ to inhibit phase segregation for enhanced hydrogen storage. *J. Phys. Chem. C* **118**, 23635−23644 (2014).

2. Arrhenius, S. A. Über die dissociationswärme und den einflusß der temperatur auf den dissociationsgrad der elektrolyte. *Z. Phys. Chem.* **4**, 96–116 (1889).

3. Chong, L., Zou, J., Zeng, X. & Ding W. Effects of La fluoride and La hydride on the reversible hydrogen sorption behaviors of $NaBH_4$: a comparative study. *J. Mater. Chem. A* **2**, 8557–8570 (2014).
